# Treatment and Prognosis of Fibrolamellar Hepatocellular Carcinoma: a Systematic Review of the Recent Literature and Meta-analysis

**DOI:** 10.1007/s11605-023-05621-z

**Published:** 2023-02-16

**Authors:** Dajana Glavas, Quoc Riccardo Bao, Marco Scarpa, Cesare Ruffolo, Zachary J. Brown, Timothy M. Pawlik, Gaya Spolverato

**Affiliations:** 1 General Surgery 3, Department of Surgical, Oncological and Gastroenterological Sciences (DiSCOG), University of Padova, Via Nicolò Giustiniani, 2, PD 35128 Padova, Italy; 2 Department of Surgery, The Ohio State University Wexner Medical Center, Columbus, OH USA

**Keywords:** Fibrolamellar hepatocellular carcinoma, Liver cancer, Hepatocellular carcinoma

## Abstract

**Background:**

Fibrolamellar hepatocellular carcinoma (FL-HCC) is a rare disease and current efforts are focused on the prognosis and on the development of efficient and specific treatments. This study aimed to review the latest evidence regarding FL-HCC treatment and prognosis.

**Methods:**

A systematic review of the literature over the past 10 years regarding FL-HCC, and meta-analysis of 1-, 3-, and 5-year overall survival (OS) comparing FL-HCC and conventional HCC were performed.

**Results:**

Overall, 1567 articles were screened, of them 21 were selected for the systematic review, and 6 for meta-analysis. Twenty-one studies included a total of 2168 patients with FL-HCC, with a median age ranging from 11 to 56 years. The majority of patients underwent surgical resection or liver transplantation. After a median follow-up ranging from 24 to 58 months, 1-year OS was 67–100% and 5-year OS was 28–65%. A total of 743 patients with FL-HCC and 163,472 with conventional HCC were included in the meta-analysis. There was a significantly improved 1-, 3-, and 5-years OS in the FL-HCC group compared to the conventional HCC group, although high heterogeneity was found. When excluding population-based studies, and including 96 FL-HCC and 221 conventional HCC patients, the heterogeneity was low, and the meta-analysis showed a significantly longer 1-year OS in patients with FL-HCC than conventional HCC; however, there were no differences at 3- and 5-years OS.

**Conclusions:**

Surgical resection for FL-HCC is currently the only curative treatment available. FL-HCC is plagued by high-recurrence rates and poor long-term outcomes which may be related to the absence of specific treatment for advanced and recurrent disease.

## Introduction

Fibrolamellar hepatocellular carcinoma (FL-HCC) is a rare primary malignancy of the liver, representing less than 1% of all malignant liver neoplasms.^1^ FL-HCC was first described in 1956 by Edmonson et al., and has unique characteristics compared with conventional hepatocellular carcinoma (HCC).^2^

Management and treatment of FL-HCC mostly overlaps those of conventional HCC. Unlike conventional HCC, which is a common malignancy worldwide, the estimated incidence of FL-HCC is reported to be 0.02 per 100,000 per year in the USA.^3^ Pathogenetic mechanisms that differentiate FL-HCC from HCC were first described by Honeyman et al. consisting of recurrent DNAJB1-PRKACA chimeric transcripts that are unobserved in cholangiocarcinoma or conventional HCC.^4^ Additionally, conventional HCC develops as a result of chronic liver disease, while FL-HCC typically arises in younger patients who do not have underlying chronic liver diseases such as viral hepatitis, fibrosis, or cirrhosis.

Like many other rare hepatobiliary malignancies, such as gallbladder cancer or cholangiocarcinoma, the rarity of FL-HCC makes understanding biological behavior and prognosis challenging. Evidence is often limited to small retrospective or population-based studies. The 5-year overall survival (OS) is approximately 80% and 10% in patients with resectable and advanced disease, respectively.^5^ Poor prognosis has been related to advanced stage at diagnosis, vague symptoms, unreliable serum tumor markers, resistance to conventional chemotherapy, and high-recurrence rates after surgery or transplant.^6,7^ Interestingly, although α-fetoprotein (AFP) is not typically elevated in FL-HCC, elevated AFP has been noted to be an independent predictor of worse survival.^8^

A standard of care has not been defined in FL-HCC due to the rarity of the disease, and the lack of studies on short and long-term outcomes related to different treatments, especially when compared to conventional HCC.^8^ Management and treatment of FL-HCC is largely based on conventional HCC experience, even if currently reliable prognostic data comparing FL-HCC and conventional HCC are lacking. Surgical resection for FL-HCC is considered a potentially curative treatment,^7,9^ with 5-year OS ranging between 52 and 62% after surgery.^10–12^ However, less than half of patients are eligible to undergo potentially curative resection. On the other hand, survival after liver transplant, liver-directed therapies, and chemo- and radiation therapy are less often reported with various outcomes.

The aim of this study is to review the most recent therapeutical advances, improvement in the management, and prognosis of FL-HCC over the past 10 years, considering all the therapeutics approaches available (surgery, transplant, chemotherapy, radiotherapy, and liver-directed therapies). Moreover, understanding that the treatment of FL-HCC is frequently guided by conventional HCC therapies, the latest improvement and long-term outcomes of FL-HCC patients will be analyzed by performing a meta-analysis of survival comparing FL-HCC versus conventional HCC.

## Methods

### Study Registration

The protocol has been registered on the International Prospective Register of Systematic Reviews (PROSPERO) (registration number, CRD42022325032) on May 31, 2022.

### Literature Search and Review

The literature search was designed to identify studies reporting clinical outcomes of patients with FL-HCC. A systematic review of PubMed, Cochrane, and Ovid archives was performed using the following Medical Subject Heading (MeSH) search terms: fibrolamellar, liver, hepatocellular. Multiple combinations of search terms were used. Articles from December 2012 to January 2022 were screened, together with the references of relevant articles. Ongoing prospective clinical trials were also included in the research (clinicaltrials.gov). The literature review was performed separately by two independent reviewers (D.G. and Q.R.B.) according to established inclusion criteria. Data regarding the year of publication, number of patients, demographics, tumor characteristics, short- and long-term outcomes, and treatment options were analyzed and registered separately by reviewers and a database of selected papers was compiled. After duplicates were removed, disagreements were settled by two additional blinded reviewers (G.S. and M.S.). The systematic review was performed according to Preferred Reporting Items for Systematic Review and Meta-Analyses (PRISMA) guidelines. Considering the design of the study (systematic review and meta-analysis), not reporting directly patients’ data, the Institutional Review Board approval was waived.

### Inclusion and Exclusion Criteria

All studies reporting clinical outcomes of patients with FL-HCC between December 2012 and January 2022 were considered eligible for inclusion. Randomized controlled trials (RCTs), controlled clinical trials, observational, retrospective, prospective, cohort, population-based, cross-sectional, and case–control studies published in the English language, which focused on survival, current strategies, and/or advances in medical or surgical therapy for FL-HCC were included. Editorials, review articles, invited commentaries and case reports, and studies with low patient numbers (< 10 cases) were excluded. Studies that included a heterogeneous population of liver neoplasms yet provided data concerning patients with FL-HCC were included. Studies with limited follow-up (less than 1 year) were excluded. Studies reporting similar cohorts of patients and overlapping populations were identified and the most recent study was considered.

### Data Extraction and Statistical Methods

Data extracted from each study were presented and analyzed using pro forma tables with pre-determined variables. Variables included were: study design, date, period, follow-up length, population demographics (number, age, sex, viral hepatitis B (HBV) and C (HCV) status, presence of cirrhosis, serum AFP, tumor dimension, presence of multifocal disease), treatment (surgery, liver transplant, liver-directed therapy [such as chemoembolization, radioembolization, radiofrequency ablation, and microwave ablation], radiotherapy, chemotherapy), and long-term outcomes (median OS, 1-, 3-, 5-year OS, disease-free survival (DFS), and recurrence rate).

A meta-analysis comparing OS in FL-HCC and HCC was performed using Review Manager 5.4 (Cochrane Collaboration, 2020). A random effects model was applied to obtain a pooled odds ratio (OR) and 95% confidence interval (95%CI) when heterogeneity was low (< 50%), whereas a fixed effect model was used if heterogeneity was more than 50%. The heterogeneity of the studies was assessed using the *I*^2^ statistic. A statistical significance was set for *p* < 0.05.

## Results

### Study Selection

**Fig. 1:**
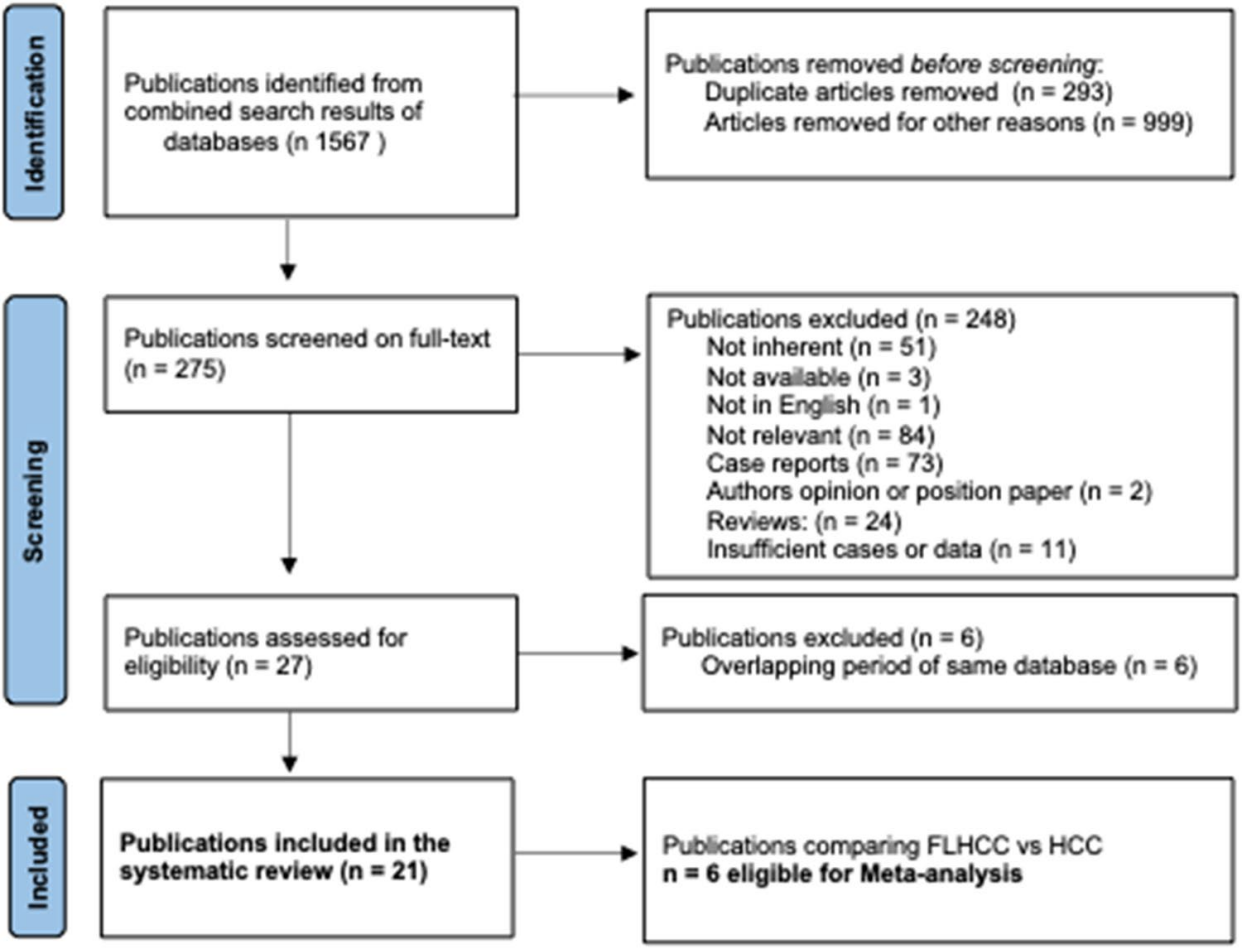
PRISMA flowchart of study selection

Overall, 1567 articles were initially screened and 275 were identified to undergo full-text review. After the removal of unavailable and not relevant articles, 21 studies were selected for the systematic review, while 7 studies reporting OS for FL-HCC and HCC were available for the meta-analysis (Fig. 1). Among the 21 included studies, 18 studies were retrospective, one study analyzed a prospectively maintained database, one study was a multicenter randomized phase II clinical trial^13^, and one study was a phase II multicenter study.^14^

### Synthesis of Clinicopathological Data

Information regarding demographics, treatment, and outcomes is shown in Table 1. The 21 studies contained a total of 2168 patients with FL-HCC; 19–67% patients were female. Median age ranged from 11 to 56 years old. Median tumor size varied from 5 to 13 cm, and 20 of 112 (18%) patients presented with multiple lesions.^10,15–17^ Seven studies reported data regarding the presence of underlying liver cirrhosis, and, out of 244 FL-HCC patients, 55(23%) had underlying liver cirrhosis, 15 (7%) had underlying HBV, and 23 (10%) had HCV.^11,12,15–19^ Serum AFP was reported in 8 studies, and was noted to be elevated (> than 15–20 or 25 ng/mL) in 199 out of 721 patients (28%).^10,15–18^

**Table 1:**
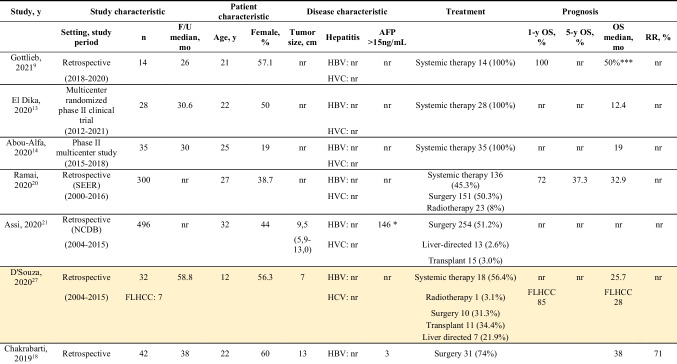

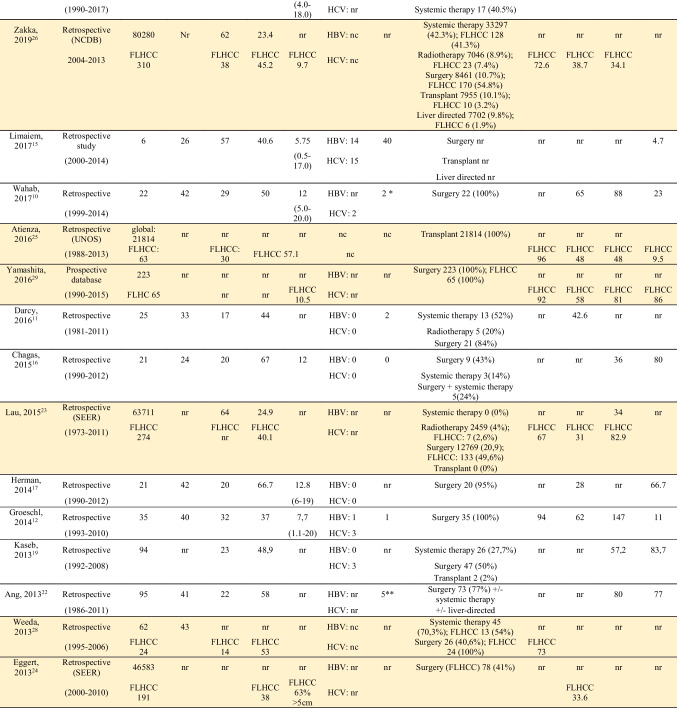
Characteristics of included studies

Studies included in meta-analysis are highlighted in yellow

*AFP

>20ng/mL

**AFP

>25ng/mL

***overall objective response

Most patients underwent surgical treatment. Sixteen studies reported outcomes data concerning surgical resection, 13 concerning chemotherapy, 6 concerning liver transplant, 5 concerning radiotherapy, and 4 liver-directed therapy. The median follow-up ranged from 24 to 58 months. One-year OS ranged from 67 to 100%, 5-year OS from 28 to 65%, with a median OS between 12.5 to 147 months, regardless of the type of treatment. OS after surgical treatment ranged from 26 to 247 months. Recurrence rate (RR), when available ranged from 4.7 to 86%.

### Review of Published Studies

Six population-based studies were included in the analysis. Ramai et al. reported data from the Surveillance Epidemiology and End Results Registry (SEER) database.^20^ The overall age-adjusted incidence of FL-HCC was 0.02 per 100,000 per year, with a bimodal age distribution between 15–19 years and 70–74 years and a median age of 27 years at diagnosis. One-half of patients underwent surgical resection, 8% radiotherapy, and 45% chemotherapy with a median survival of 32.9 months and a three times higher risk of death for patients not treated surgically. Assi et al. reported data from the National Cancer Database (NCDB).^21^ In this study, 496 patients were diagnosed between 2004 and 2015 with a median age of 32 years, and 90% of patients underwent surgical resection. An overall median OS of 68.5 months was reported, and a worse OS was found in patients treated with adjuvant or neoadjuvant chemotherapy than patients without systemic therapy (22 vs. 42.8 months, respectively).

As for medical therapy, a retrospective review of 22 patients with advanced, relapsed, or refractory FL-HCC treated with 5-fluorouracil (5-FU), interferon and nivolumab demonstrated a 50% objective response rate (ORR) and 93% tumor control rate, identifying this combination as a promising option to reduce disease progression.^9^ On the contrary, there was no improvement in outcomes in a multicenter randomized phase II study of estrogen deprivation combined with an mTOR inhibitor for unresectable FL-HCC, reporting a 12.4-month OS.^13^ Furthermore, a phase II multicenter study by Abou-Alfa et al. assessed the utility of an oral anti-Aurora kinase A (AURKA) inhibitor in the treatment of 35 patients with advanced FL-HCC.^14^ This study failed to meet its primary efficacy endpoint with an OS and PFS of 3.9 and 19 months, respectively.

Several retrospective studies reported results on treatment, outcomes, and risk factors associated to poor survival. Chakrabarti et al. reported a 5-year OS of 86% for patients with stage I disease, 44% for patients with stage II to IVB disease, and a median OS of only 10 months for patients with unresectable disease.^18^ Wahab et al., Herman et al., and Chagas et al. described the outcomes of 29, 21, and 21 patients, respectively, with a 5-year OS of 65%, 28%, and a median OS of 36 months, respectively.^10,16,17^ All studies reported vascular invasion as a negative prognostic factor. Additionally, macrovascular invasion, unresectable disease, lymph node metastases, advanced stage, and incomplete resection were associated with poor survival, as described by Darcy et al., Kaseb et al., and Ang et al.^11,19,22^ Moreover, a retrospective study by Groeschl et al. examined a cohort of 30 patients treated with curative resection and 5 patients underwent palliative operations.^12^ For patients undergoing curative-intent surgery, 5-year OS and recurrence-free survival (RFS) were 62% and 45%, respectively.

### Comparative Studies of Patients with FL-HCC and Conventional HCC

Seven studies compared the characteristics, treatments, and outcomes of FL-HCC and conventional HCC. SEER database studies by Lau et al. and Eggert et al. reported data on outcomes, demographics, and treatment of 63,711 HCC (274 of which were FL-HCC) and 46,583 HCC (191 FL-HCC) patients, respectively.^23,24^ Overall, FL-HCC had a higher mean OS when compared to conventional HCC. Lau et al. reported a median age of 64 years at diagnosis and a male prevalence of 75.1%.^23^ In all patients with HCC, treatment consisted of surgery alone in 20.9% of patients, radiotherapy alone in 4% of patients, and a combination of surgery and radiation in 0.5% of patients; 45,558 patients (74.5%) were untreated. Mean actuarial survival was higher for pediatric patients. Of the 274 patients with FL-HCC, 49.6% underwent surgery as the primary treatment modality, and a combination of surgery and radiation was utilized in 2.6% of cases. Mean OS after surgery alone was 11.3 years, compared to 7.4 years for combination treatment, and 1.9 years for patients undergoing no treatment. Eggert et al. describe a cohort of 46,392 HCC and 191 FL-HCC patients diagnosed between 2000 and 2010.^24^ There was a male prevalence for all histologic types and a larger tumor size in FL-HCC cases. Surgical resection was the preferred treatment modality and FL-HCC patients experienced an increased 5-year relative survival over conventional HCC. No difference in 5-year OS (51% and 57% for HCC and FL-HCC respectively) was noted among patients undergoing curative-intent treatment.

Atienza et al. analyzed data from the United Network for Organ Sharing (UNOS) database between 1988 and 2013 and identified 63 patients with FL-HCC and 21,751 patients with HCC who underwent a liver transplant.^25^ FL-HCC usually arose in healthy liver patients, where only 1 FL-HCC patient had underlying HCV infection, while 3976 and 686 HCC patients had HCV or HBV infection respectively. Additionally, 441 HCC patients presented underlying steatohepatitis. One-, 3-, and 5-year OS were 96%, 80%, and 48% respectively in FL-HCC, and 86%, 73%, and 64% in HCC.

Zakka et al. queried the NCDB between 2004 and 2013 collecting data on 78,461 conventional HCC and 310 FL-HCC patients.^26^ As far as FL-HCC treatment was concerned, six patients underwent ablation, 170 patients underwent surgical resection, and 10 patients underwent liver transplant; moreover, 23 patients were treated with radiation and 128 patients with chemotherapy. Among patients with HCC, 7696 patients were treated with ablation, 8291 with surgery resection, 7945 with a transplant, 7023 underwent radiation, and 33,169 chemotherapy. Median OS was 34.1 and 13.4 months for FL-HCC and HCC respectively. One- and 5-year OS was 72.6 and 38.7% respectively for FL-HCC, and 52.2 and 22.6% for conventional HCC.

Two retrospective studies by D’Souza et al. and Weeda et al. compared outcomes and characteristics of FL-HCC and conventional HCC.^27,28^ The study by D’Souza et al. contained 32 patients treated between 2004 and 2015, 7 of them had FL-HCC.^27^ Treatment options included liver transplantation in 11 patients, hemi-hepatectomy in 9 patients, segmentectomy in 1 patient, and medical therapy in 18 patients (13 received neoadjuvant and 5 adjuvant therapy). Outcomes were assessed after a median follow-up of 58.8 months. They found a 43.8% global survival, 28% for FL-HCC, and 48% for conventional HCC. Weeda et al. reported on 38 patients with HCC and 24 patients with FL-HCC.^28^ Patients were treated either with primary surgery: 8 (33%) FL-HCC and 5 (13%) HCC patients, or with chemotherapy: 13 FL-HCC (54%) and 32 HCC (84%) patients. No statistically significant difference emerged at 3-year follow-up suggesting comparable long-term outcomes.

In addition, a prospectively study by Yamashita et al. between 1990 and 2015 collected data from 65 surgically resected non-cirrhotic FL-HCC patients and 158 non-cirrhotic HCC patients.^29^ No patients with FL-HCC had underlying viral hepatitis, while 25% of conventional HCC patients had viral hepatitis. Multiple tumors were present in 20% of FL-HCC cases and 22% of HCC cases, and median follow-up varied between 48 and 52 months. All patients were treated with surgical resection: 74% of FL-HCC and 59% of HCC underwent major hepatectomy. Five-year OS and median OS were similar in the two groups (67% vs. 58% and 137 and 81 months in HCC and FL-HCC respectively). Recurrent disease was observed in 86% of patients with FL-HCC. RFS was significantly longer in HCC (5-year RFS 55% vs. 10%, median RFS 108 vs. 11 months).

### FL-HCC and HCC Survival Comparison: Meta-analysis

**Fig. 2:**
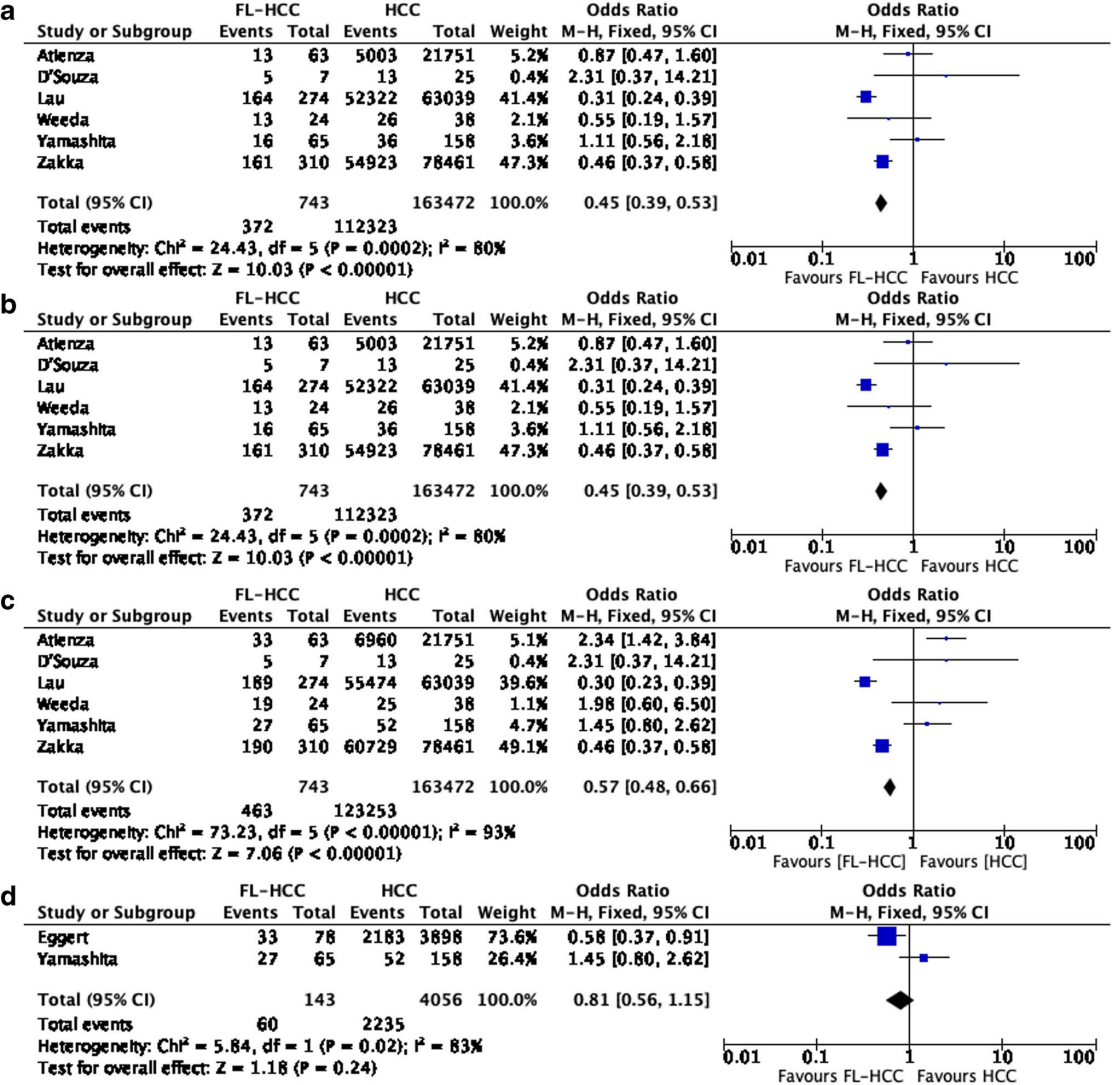
Meta-analysis of overall survival; **a** 1-year OS, **b** 3-years OS, **c** 5-years OS, **d** 5-years OS in resected patients

Out of seven studies reporting comparative survival analysis, 6 studies, including 3 retrospective studies and 3 population-based studies, were included in the meta-analysis. One study was excluded for potential overalapping study cohort (SEER database); however, this study was included in surgical resected patients’ meta-analysis.^24^ Thus, a meta-analysis comparing OS for FL-HCC and HCC was performed. Overall, when including all studies, a total of 743 patients with FL-HCC and 163,472 with HCC were included. There was a significantly improved 1-, 3-, and 5-years OS in FL-HCC group compared with the HCC group (OR 0.31 95% CI 0.22–0.43, *p* = 0.09; OR 0.45 95%CI 0.39–0.53, *p* < 0.00001; and OR 0.57 95% CI 0.48–0.66, *p* < 0.00001) (Fig. 2), although high heterogeneity was found.

Two studies compared 5-year OS specifically in surgically resected patients, including 143 surgically resected patients with FL-HCC and 4056 for conventional HCC. Overall, no difference in OS was observed between groups (OR 0.81 95%CI 0.56–1.15, *p* = 0.24) (Fig. 2d).

**Fig. 3:**
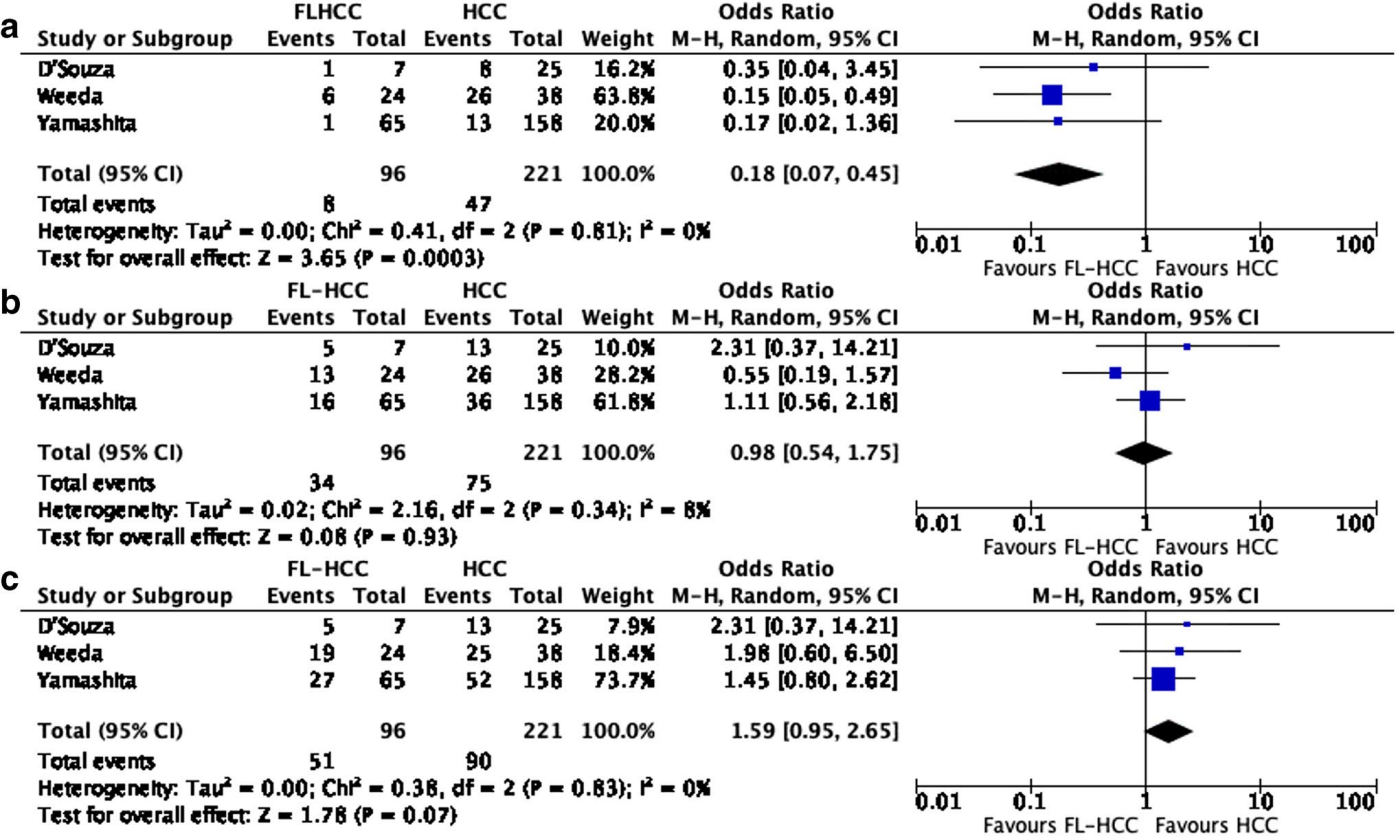
Meta-analysis of overall survival including retrospective studies only; **a** 1-year OS, **b** 3-years OS, **c** 5-years OS

When considering only retrospective studies, three studies were included in the meta-analysis consisting of 96 patients with FL-HCC and 221 patients with HCC. There was a significantly longer 1-year OS in patients with FL-HCC vs. HCC (OR 0.18 95%CI 0.07–0.45, *p* = 0.0003), whereas there was no difference in 3- and 5-year OS (OR 0.98 95%CI 0.54–1.75, *p* = 0.93; and OR 1.59 95%CI 0.95–2.65, *p* = 0.07) even if there was a trend toward benefit in HCC in 5-year OS (Fig. 3). The heterogeneity of these studies was low.

## Discussion

FL-HCC is a rare primary liver malignancy, and it is often difficult to study and parse out the similarities and differences compared with traditional HCC. In the current study, FL-HCC was confirmed to be a rare tumor, and there is a lack of knowledge on the impact of different treatment modalities and prognosis in patients afflicted by FL-HCC. Currently, the only curative treatment for FL-HCC is surgical resection, whereas only a few studies report on liver transplantation for FL-HCC, since liver transplant is primarily used in end-stage liver disease. In patients with unresectable disease or outside transplant criteria, chemotherapy, radiotherapy, and liver-directed therapies may have a potential role, but data on their impact on survival and recurrence for patients with FL-HCC is limited.

FL-HCC was confirmed to affect young patients, diagnosed more often at an advanced stage, and almost 20% of patients had multiple lesions. Unlike HCC, FL-HCC was found to arise in healthy livers, but 20% of patients presented underlying liver cirrhosis. Additionally, in 8 studies AFP was reported to be elevated in 28% of cases. Recently, a population-based study by McDonald et al. investigated the factors associated with survival in FL-HCC patients.^8^ Multiple tumors, elevated AFP, and regional lymph node metastases were independently associated with worse survival. A median OS of 134 months was noted in patients with normal AFP vs. 43 months in elevated AFP patients.^8^ AFP is utilized as a prognostic biomarker for HCC, but its prognostic role in FL-HCC is less clear. AFP may represent a useful tumor biology marker to identify biological aggressive FL-HCC, and high-risk patients.

Surgical resection remains the only curative treatment available for stage I–III FL-HCC. However, only half of the patients in the reported studies underwent curative-intent surgical resection. Eggert et al. reported that patients treated with curative intent, including resection, transplant, and radiofrequency ablation, had a reported 5-year OS of 56–58%.^24^ A retrospective analysis from MD Anderson Cancer Center (MDACC) on resected FL-HCC reported a higher stage at diagnosis, a higher rate of lymph node metastases, and a greater rate of major hepatectomy, when compared with conventional HCC.^29^ When considering only stage I–III disease, the OS of FL-HCC and HCC was similar, whereas RFS was significantly longer in conventional HCC. Recurrence occurred in most of the patients after resection (86%). The only predictors of recurrence were vascular invasion and age > 25. Similarly, patients with conventional HCC have high-recurrence rates with up to 70% of patients experiencing recurrence at 5 years. However, unlike patients with conventional HCC, patients with FL-HCC have no underlying liver disease, which may make cause different patterns of recurrence compared to conventional HCC. According to previous studies, it was not possible to clarify if the high rate of recurrence was related to aggressive biology, advanced disease at diagnosis, or other unknown factors. Additionally, there is no current adjuvant therapy approved for patients with HCC.^30^

In the meta-analysis of all studies comparing FL-HCC and HCC, patients with FL-HCC had improved OS compared to patients with HCC. As the heterogeneity of the studies was high, and the results were influenced by the population-based studies,^23,24,26^ we also performed a separate meta-analysis excluding population-based. In the population-based studies, patients had improved survival with FL-HCC versus HCC. The only study that supported an improved survival in HCC was the study by Atienza et al., which included patients treated with a liver transplant.^25^ In this study, the 5-year OS was 48% and 68% in FL-HCC and HCC respectively, with a reported rate of graft survival of 48 and 64%. The authors suggested that a decreased 5-year OS in patients with FL-HCC was due to more advanced disease at the time of transplant. This trend is confirmed in our meta-analysis including retrospective and prospective studies. The 1-year improved OS in FL-HCC was no longer present at 3 and 5 years. The improved 1-year OS in FL-HCC could be explained considering the impact of surgery for resectable disease and the fact that FL-HCC occurred in normal liver, while conventional HCC more often occurred in chronic liver disease, and short-terms outcomes could be influenced by post-operative liver failure. In the meta-analysis by Njei et al., including 11 studies involving 289 FL-HCC and 9571 conventional HCC patients, an improved 5-year OS for FL-HCC compared to HCC was reported. ^31^ However, when limiting the analysis to 3 studies in non-cirrhotic patients, no difference was found between FL-HCC and HCC. The author concluded that the apparent survival benefit in FL-HCC reported in the previous studies may be related to chronic liver disease in HCC. By updating this analysis, considering the last improvement in the management of chronic liver disease, our data support no differences in terms of 5-year OS when considering retrospective studies only, which included mostly patients treated with surgery, which remained the only effective treatment.

Conventional HCC is the most common primary liver malignancy and treatment options are still evolving. Only a few studies considered the role of non-surgical treatment in FL-HCC, which may find a role in the treatment of advanced and/or recurrent diseases. Several chemotherapy regimens have been investigated in the treatment of FL-HCC such as fluoropyrimidine, interferon, doxorubicin, cisplatin, gemcitabine, oxaliplatin, bevacizumab, and sorafenib. El Dika et al. randomized 28 patients with unresectable FL-HCC to receive everolimus, letrozole + leuprolide, or both.^13^ There was no survival benefit reported with these therapies. In a phase II study, Abou-Alfa et al. did not find a survival benefit using oral anti-aurora kinase A.^14^ The only study reporting a survival benefit was a retrospective analysis by Gottlieb et al. that included 14 patients treated with 5-FU, interferon, and nivolumab with promising results in high-risk FL-HCC.^9^ The immunochemotherapy demonstrated a tumor control rate of 93% with an ORR of 50% in recurrence and refractory FL-HCC. The MDACC is recruiting patients with unresectable FL-HCC using the same therapeutic combination (NCT04380545). Other ongoing trials are enrolling patients to evaluate the role of immunotherapy, such as pembrolizumab, in FL-HCC (NCT04134559). Both trials are enrolling patients with FL-HCC and HCC, although it is assumed that FL-HCC and conventional HCC are two distinct entities, requiring specific treatment. Johns Hopkins University is enrolling FL-HCC patients to assess the safety and tolerability of targeted therapy (DNAJB1-PRKACA fusion kinase) in combination with nivolumab and ipilimumab (NCT04248569). As more is learned about the underlying mechanisms of disease and differences between conventional HCC and FL-HCC more novel therapies, such as targeted therapies or immunotherapies, may be developed to improve survival in patients with FL-HCC. Unfortunately, current data on FL-HCC genomics are lacking, and only DNAJB1-PRKACA gene fusion has been systematically reported.

Several limitations of this study should be discussed. This study is a systematic review of the recent literature about FL-HCC, and due to the rarity of the disease, there is not a wide availability of published series. Most of the published studies have a small sample size and underpowered results. For this reason, most of the recent literature is based on the availability of databases and registries (NCDB, SEER, UNOS). Additionally, there is a lack of multicenter studies or registries that may increase sample sizes and improve data collection and reporting for this rare disease. Memorial Sloan Kettering Cancer Center is creating an FL-HCC registry to collect clinical information to improve knowledge of FL-HCC (NCT04874519). The meta-analysis also included small-number studies, and their results are rather heterogeneous, as well as studies covering a wide timespan (more than 30 years). Even when excluding population-based studies, which limits the heterogeneity of the studies, the included studies reported different treatment paradigms often without specifying treatment details, such as surgical resection, chemotherapy, liver transplant, or combined approaches, in small cohorts. Many studies did not distinguish between FL-HCC and conventional HCC when evaluating the long-term outcomes relative to different treatments. Furthermore, considering the early onset of the FL-HCC, it would be also important to consider DFS. Data regarding DFS are lacking in the included studies, and a meta-analysis of DFS was not possible. However, the results of our meta-analysis support that HCC and FL-HCC are different diseases. As such, they require different treatment, especially when advanced and recurrent disease occurs a time beyond the scope of surgery when novel or targeted therapies are required.

## Conclusions

FL-HCC is a rare neoplasm, and surgical resection is currently the only curative treatment available. FL-HCC is plagued by high-recurrence rates and poor long-term outcomes which may be related to the absence of specific treatment for advanced and recurrent disease. Further studies are required to evaluate the effect of specific treatment for FL-HCC.
